# Can the anatomy of abnormal flowers elucidate relationships of the androecial members in the ginger (Zingiberaceae)?

**DOI:** 10.1186/s13227-020-00157-8

**Published:** 2020-06-09

**Authors:** Xiumei Li, Tian Fan, Pu Zou, Wenhu Zhang, Xiuju Wu, Yixin Zhang, Jingping Liao

**Affiliations:** 1 Guangdong Provincial Key Laboratory for Crop Germplasm Resources Preservation and Utilization, Agricultural Biological Gene Research Center, Guangdong Academy of Agricultural Sciences, Guangzhou, 510640 China; 2 School of Life Science, Guangzhou University, Guangzhou, 510006 China; 3 Key Laboratory of Plant Resources Conservation and Sustainable Utilization, South China Botanical Garden, Chinese Academy of Sciences, Guangzhou, 510650 China; 4 College of Agronomy, Hunan Agricultural University, Changsha, 410128 China

**Keywords:** Zingiberaceae, Vascular, Androecial member, Abnormal flower

## Abstract

**Background:**

Interpretation of the floral structure of Zingiberaceae has long concentrated on the relationships of the androecial members. It suggested that labellum is composed of two structures rather than three or five, and glands are interpreted either as gynoecial part or as androecial members.

**Methods:**

Serial sections were used to observe the vasculature of normal and two-staminate flowers in *Alpinia intermedia* ‘shengzhen’. Floral diagrams were drawn to interpret the morphological category of the floral organs and the relationships of the androecial members. Androecial vascular bundles were associated with carpellary dorsal bundles (CDBs) and parietal bundles (PBs) in a Zingiberales phylogeny setting using ancestral state reconstruction.

**Results:**

Anatomical observations demonstrate that the fertile stamen(s) incorporate parietal bundles both in normal and two-staminate flowers. The three appendages represent the three members of the outer whorl of the androecium, while the labellum represents the inner whorl of the androecium in the two-staminate flower. Reconstruction of the origin of the vascular system in the androecium suggests that the outer whorl of androecium receives its vascular supply from the CDBs, and the inner whorl of androecium receives from the PBs in both the basal banana group and the more derived ginger clade.

**Conclusions:**

The present study adds to a growing body of literature suggesting that the anatomy of abnormal flowers may not provide enough evidence for elucidating the relationships of the androecial members, and help us to better understand how the vascular system is constructed during the androecial petaloidy evolution.

## Background

Interpretation of the flower has long concentrated on the relationships of the androecial members in Zingiberales, which is divided into the Banana Group (Musaceae, Strelitziaceae, Heliconiaceae and Lowiaceae (Orchidantha)) and the Ginger Group (Zingiberaceae, Costaceae, Marantaceae, and Cannaceae) [29, 30]. The androecium is acknowledged to typically consist of two whorls, which possess three members each. In most flowers of the Banana Group, one fertile stamen is either replaced by a staminode or is completely absent, with five functional stamens remaining [55]. Except for the Heliconiaceae family, which has a staminode of the outer whorl opposite the adaxial sepal, the other three families have a staminode of the inner whorl opposite the adaxial petal. In the Ginger Group, by contrast, the flowers are highly modified compared to the idealized monocotyledon; five fertile or more stamens are transformed into petaloid structures, leaving only one (Zingiberaceae, Costaceae) or one fertile half stamen (Marantaceae, and Cannaceae). This single (or half) fertile stamen is always the same member of the inner androecial whorl. Thus homeosis has played an important role in the floral evolution of the ginger group, and these sterile stamens share positional homology with stamens in the banana group and other monocots [3, 25].

Homeosis is “the assumption by one part [of an organism] of likeness to another part” [4]. Leavitt [32] accepted and perfected the term by describing the phenomenon of homologous heteromorphism in many plants. If the degree of likeness is very high, one organ type is directly replaced with another in a 1:1 substitution. If, however, the degree of likeness is lower, an organ intermediate between two normally distinct organ categories develops [57]. One can discover morphologies that correspond more or less to some of the homeotic mutants in many plant species [57]. As early as 1760, Caspar Friedrich Wolff believed that different flower organs could be transformed and equal to each other within an idealist morphological perspective. About 20 years later, a similar theory was proposed by Johann Wolfgang Goethe based on the observation of serial abnormal flowers [63]. Abnormal flowers provide opportunities to study of homeosis from the view of comparative anatomical morphology. There are a series of cases of abnormal flower in Zingiberales. In Musaceae, for example, a decreased number of stamens in the abnormal flowers of the Dwarf Cavendish banana are the result of the adnation of stamens to the style and the arrested development of some of the stamens which remained dwarf and staminodial [14]. Many of these “abnormal” or “mutant” phenomena have been recognized as homeosis in Cannaceae, they represent a biological phenomenon that caused either by genetic differences (genotypic variation) or by the effect of environmental factors on the expression of the genetic potentials (phenotypic variation), which is important for evolution [39, 66]. The abnormal flowers have also been reported in Zingiberaceae, especially in the genus *Alpinia*; e.g., only two loculi in lateral positions in *Hedychium coronarium*, two stamens and one sterile appendage in *Alpinia vittata* [47]. Maas [37] found an abnormal flower of *Renealmia goyazensis*, in which two fully developed anthers replaced the lateral appendages of the labellum. Song et al. [59] reported a considerable diversity of developmental transformations in the genus *Alpinia*. Analyses of the abnormal flowers allow us to deduce robust rules of homeotic transformation that could potentially be generalized across the Zingiberaceae. Floral anatomy, in terms of vasculature for an evolutionary relationship, was under suspicion for failing to provide clear details and valid lines of evidence [10]. However, because of the nebulous and reticulate relation between evidence and phylogeny, not only anatomists but all others have yet to establish objective and useful clues that can solve phylogeny without any ambiguity [43], and that the existence of vascular connections and the development of primordia facilitate distinguishing a staminodial structure [12]. Therefore, the significance of anatomical characters should not be downgraded. Comparing of floral anatomical characters between normal and abnormal would contribute to the understanding of homeosis on the floral construction.

**Fig. 1:**
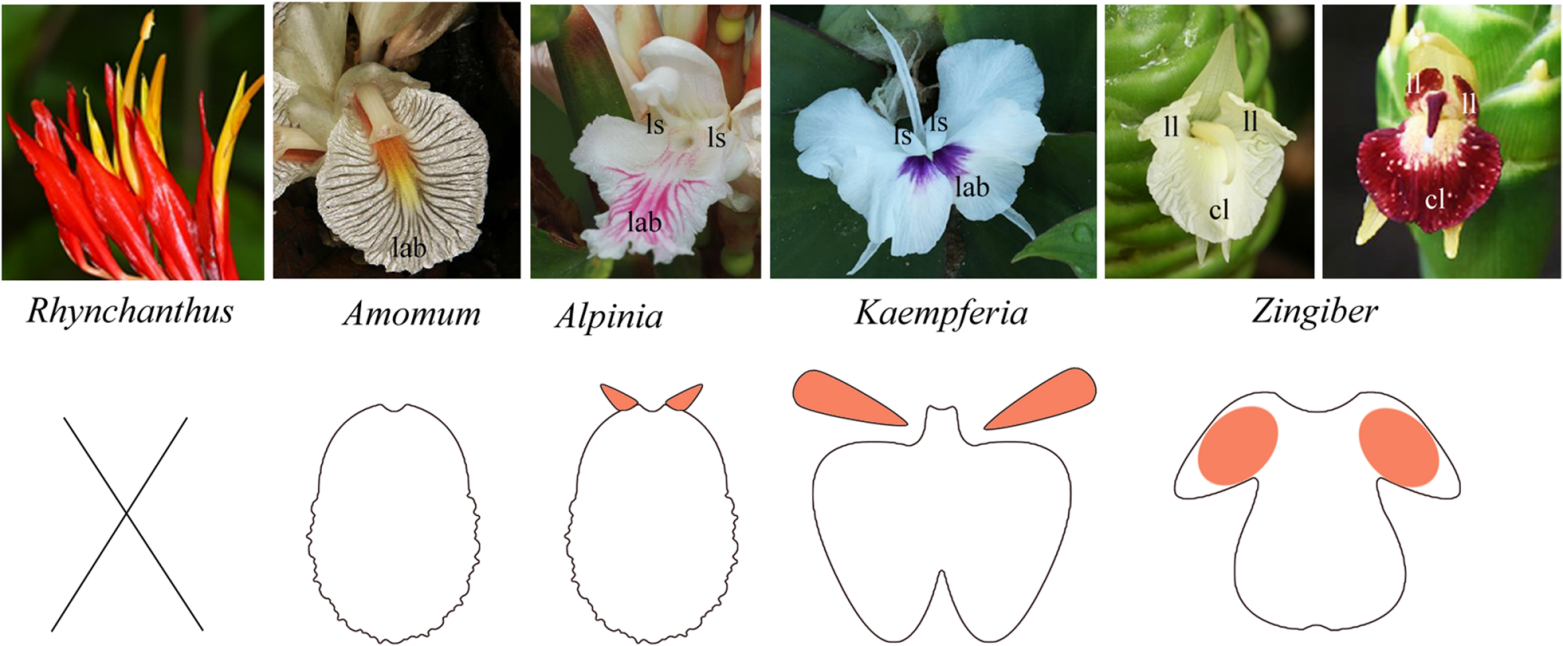
Three typical types of labellum in Zingiberaceae. Representative genera of each category are listed in the figure above. Five stamens are completely absent, resulting in not any novel structure in *Rhynchanthus* genus. The lateral appendages of unlobed labellum correspond to the petaloid of bilobed labellum and the lateral lobe of trilobed labellum, which we marked in orange color. Lab, labellum; ls, lateral staminode; cl, central lobe; ll, lateral lobe

In Zingiberaceae, unlike six stamens arranged in two whorls, putative 2 to 5 staminodes fuse in varieties of combinations to form a novel structure—staminodial labellum [25]. The labellum displays as an unlobed structure with/without two lateral appendages at the base (e.g., *Elettariopsis, Alpinia*), or a trilobed structure adanated by two lateral segments (e.g., *Zingiber*), or a bilobed structure with two lateral staminodes petaloid (e.g., *Globba*, *Kaempferia*, most *Hedychium*); and to the extreme, not any novel structure exists in *Rhynchanthus* genus (Fig. 1a). The organ identity of the labellum is considered to be associated with the androecium member, and its relationship to glands has received contrasting interpretations (Fig. 1b). Brown [8] regarded the outer androecial whorl is represented by the labellum and the two lateral staminodes (if exist), while the inner androecial whorl is represented by the posterior stamen and the two glands. In Payer’s [42] perspective, based on the trimerous plan of floral construction, the labellum is a double structure. He interpreted the outer androecial whorl to be represented by the two lateral staminodes, and the anterior member of this whorl was missing. The inner androecial whorl is composed of the posterior stamen and the labellum; the latter is derived by the congenital fusion of two lateral staminodes. Gregory [17] proposed the labellum as a triple structure: the central portion of the labellum belongs to the outer androecial whorl, while its lateral portions belong to two members of the inner androecial whorl. Thompson [65] agreed with the “triple structure” by ontogenetic observations but of a different derivation. In his description, labellum was developed from primordia 8, 11, and 13 of a spiral sequence, and the epigynous glands were called stylodes. However, Payer’s “double structure” interpretation on labellum was most widely accepted recently, followed by Kirchoff’s [24, 25, 27] more detailed ontogenetic work. In Kirchoff’s observation, the labellum is generated by connecting the primordia of the two inner petaloid staminodes of intercalary growth, and the outer androecial staminode initiated between the two primordia ceases growth shortly after the labellum initiating. Following recent developments in both phylogenetics and developmental genetics of Zingiberaceae and related families, we survey the anatomy on the vasculature of normal flower and abnormal, two-staminate flower in *Alpinia intermedia* ‘shengzhen’, aim to provide new evidence to evaluate the morphological category of the floral organs. We also intend to assess if the anatomy of abnormal flowers could elucidate relationships among the androecial members in the Zingiberaceae. Last but not least, we summarized floral vasculature characters, based on previous anatomical studies in the eight families of Zingiberales, to better understand how the vascular system is constructed during the androecial petaloidy evolution. The origin of the vasculature of normal and abnormal flowers may play an important role in morphological character mapping for assessing floral homologies, especially in relationships of androecial members.

## Materials and methods

### Plant materials

Living materials of *Alpinia intermedia* ‘shengzhen’ were collected at the Ginger Garden of South China Botanical Garden (SCBG) in Guangzhou, Chinese Academy of Sciences. Mr. Ye Yushi provided the materials and plant identification. Both of the normal flowers with an accession number 20150507 and the abnormal flower that has been isolated without accession number are located in SCBG. The vouchers are preserved in the South China Botanical Garden Herbarium (Additional file 1: Table S3).

### Paraffin section

Flowers of young inflorescences were immersed in formalin–acetic acid–alcohol (FAA) and were vacuumed to allow FAA to penetrate the tissues. The samples were fixed in FAA for at least 48 h and then preserved in 70% ethanol, as described previously. Briefly, three to five fully developed flowers were selected and dehydrated and then embedded in paraffin. Transverse serial sections of the floral parts, ovary prolongation, locular region, and pedicel were cut at 8–12 um using a microtome and mounted on glass slides before staining with Ehrlich’s hematoxylin. The terminology here followed Rao [46–52] and Liao et al. [36]. Adaxial/abaxial was used to describe the position of floral organs with respect to the inflorescence axis at stages of floral development. The slide specimens are preserved in the Key Laboratory of Plant Resources Conservation and Sustainable Utilization, SCBG.

### Reconstruction of the phylogeny of Zingiberales

To obtain the phylogenetic relationships of these taxa for floral vascular trait analysis, we reconstructed phylogenetic trees. Our sampling focused on Zingiberales to collect data with representatives from each of the eight families, in addition to the outgroup. We selected the nuclear ribosomal internal transcribed spacers 1 and 2 (ITS) and the external transcribed spacer (ETS), the plastid markers trnL-F, and trnL-rpl32, introns from the putatively single copy nuclear genes *calmodulin* (*CaM*) based on recently publications [2, 19]. Nucleotide sequences downloaded from Genbank (https://www.ncbi.nlm.nih.gov/genbank/) were aligned using MUSCLE version 3.5 [13] implemented in BioEdit v.7.0.0 [18], and then adjusted manually. Individual loci trees were evaluated using RAxML, but result in poor support for topological relationships due to the absent sequence information of some species. We increased sampling especially for branches with low supporting rate, *AGL6* MADS-box gene which putatively single copy or loss the copies [71] was selected to generate a combined matrix.

Best-fit substitution models for each dataset were selected under the Bayesian Information Criterion (BIC) using MEGA6, the model used was the GTR + G for nrITS, K2 + G+I for nrETS, T92 + G for CAM, T92 + I for TRN, GTR + G+I for RPLN, GTR + G for AGL6. To access potential substitution saturation, we calculated pairwise model-corrected distances rates of transitions and transversions on all nucleotide sides in MEGA6 [31], and exported the data to graph. The pairwise deletion on gaps/missing data treatment yielded more data than complete deletion, but the results were similar. Pairwise GTR-corrected distances were also plotted against rates of transitions and transversions for fully resolved sites and all positions combined in DAMBE [70]. For sequences containing unresolved bases, we dealt with them as “?”. If the test statistic is significantly lower than the critical value, then there is no significant saturation; if not, there is substantial saturation or the sequences are poor for phylogenetics. The phylogenetic relationships were conducted for the five markers using both maximum likelihood (ML) and Bayesian inferences (BI) by RAxML version 8.2.0 [62] and MrBayes version 3.2.6 [53], respectively, on the CIPRES Science Gateway [40]. The ML phylogeny was inferred under the GTRGAMMA model, and support for the branches was estimated from 1000 bootstrapping replicates under GTRCAT. The Bayesian inferences were performed with Markov chain Monte Carlo (MCMC) running with four chains each for 1000,000 generations. Trees were sampled every 1000 generations. The specific model for each dataset suggested by MEGA6 was applied to the Bayesian analyses. Convergence of the four MCMC chains was assessed by checking that the average standard deviation of the split frequencies dropped below 0.01. The first 25% of the trees were discarded as burn-in, and 75% of remaining trees were used to reconstruct the majority-rule consensus tree. Finally, a phylogeny was carried out manually using Mesquite version 3.5 [38]. This phylogeny lacking branch length parameter was used for parsimony-based ancestral state reconstruction.

### Trait evolution for the androecial whorl

To achieve an understanding of the evolutionary transition in Zingiberales androecial whorl morphology and vasculature, we selected and analyzed the performance of PBs, CDBs, and vascular plexus characters in androecial members within the families. We collected information on floral vasculature from the literature. Outgroup taxa from commelinid monocot orders were selected in terms of the previously published phylogenies [2]. Mesquite was used to build the vascular data matrix and optimize characters on the ML tree based on the combined data set. Character traits and their state matrices were coded as follows: inner androecial whorl receives strands from 0, undetermined; 1, PBs; 2, CDBs and PBs; 3, vascular plexus formed by CDBs and PBs with other branches. The outer androecial whorl is supplied by 0, undetermined; 1, CDBs; 2, CDBs and PBs; 3, vascular plexus formed by CDBs and PBs with other branches.

## Results

### Floral morphology

**Fig. 2:**
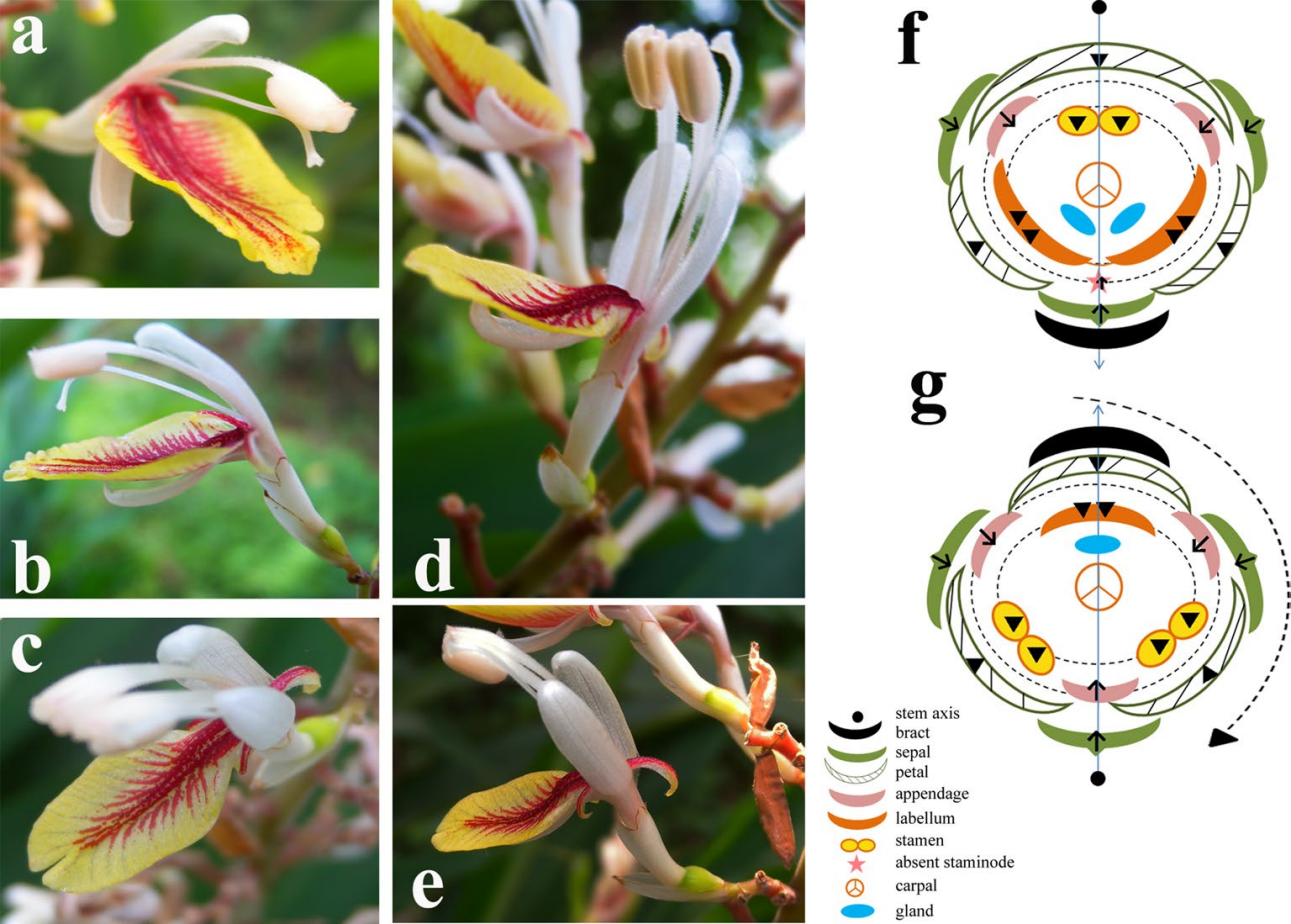
Normal and two-staminate flowers of *Alpinia intermedia* ‘chenshengzhen’. **a**, **b** Normal flower; **c**–**e** two-staminate flower; **f** floral diagram of normal (upper) and two staminate flower (lower), diagram of normal flower based on observations by Kirchoff et al. [22–25, 28], Kress [29], Bartlett and Specht [3], and diagram of the two-staminate flower based on our study. Lines with arrows indicate the planes of floral symmetry. Dotted circles indicate the inner and outer androecial whorls. Arrow (↑), carpellary dorsal bundles (CDBs); arrowhead (▼), parietal bundles (PBs); placental bundles (PLBs)

Normal flowers are bisexual and zygomorphic, calyx tubular, split on one side, formed by 3 sepals. Corolla tubular with three lobes, 1 adaxial wider than 2 abaxial. Stamen 1, adaxial, with two thecae. Lateral appendages 2, subulate, adnate to base of the abaxial labellum (Fig. 2). Ovary with 3 locules. Style 1. The two-staminate flowers are different in that the fertile stamens are 2, subulate appendages alternated with stamens and labellum are 3, and three petals are opposite to stamens and labellum, respectively (Fig. 2). The two types of flowers are arranged on the same inflorescence.

In the two-staminate flower, the gland is smaller than the usual one, and the labellum is much smaller than that in the normal flower. The development of external organs has an influence on the next organs that will develop; the presence of three outer staminodes has the effect that the labellum is much smaller. It is inferred that the more the primordia there are, the more space they occupy on the floral meristem at floral development, thus reducing the space available for subsequent organs to be initiated. Two adaxial petals embrace the abaxial petal. Two adaxial petals are set against the two fertile stamens, while the abaxial one against the labellum. So the position of the two fertile stamens and the labellum are anti-petalous.

### Floral vasculature of the normal flowers and the two-staminate flowers

**Fig. 3:**
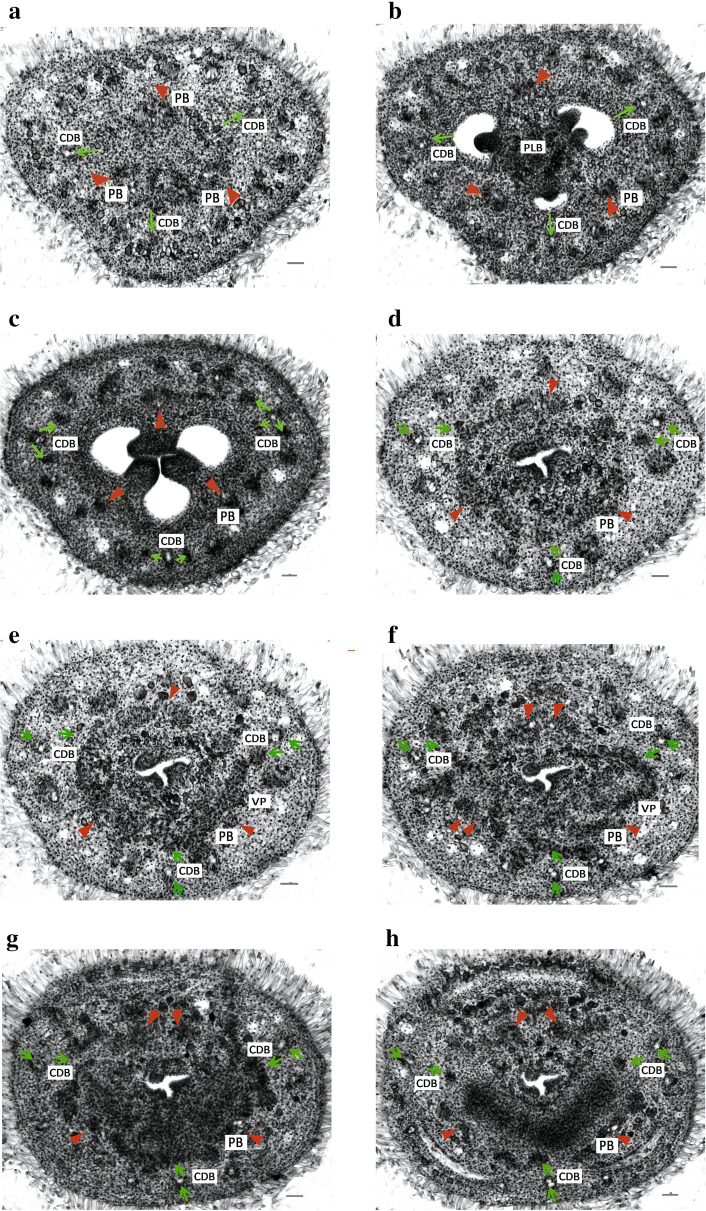

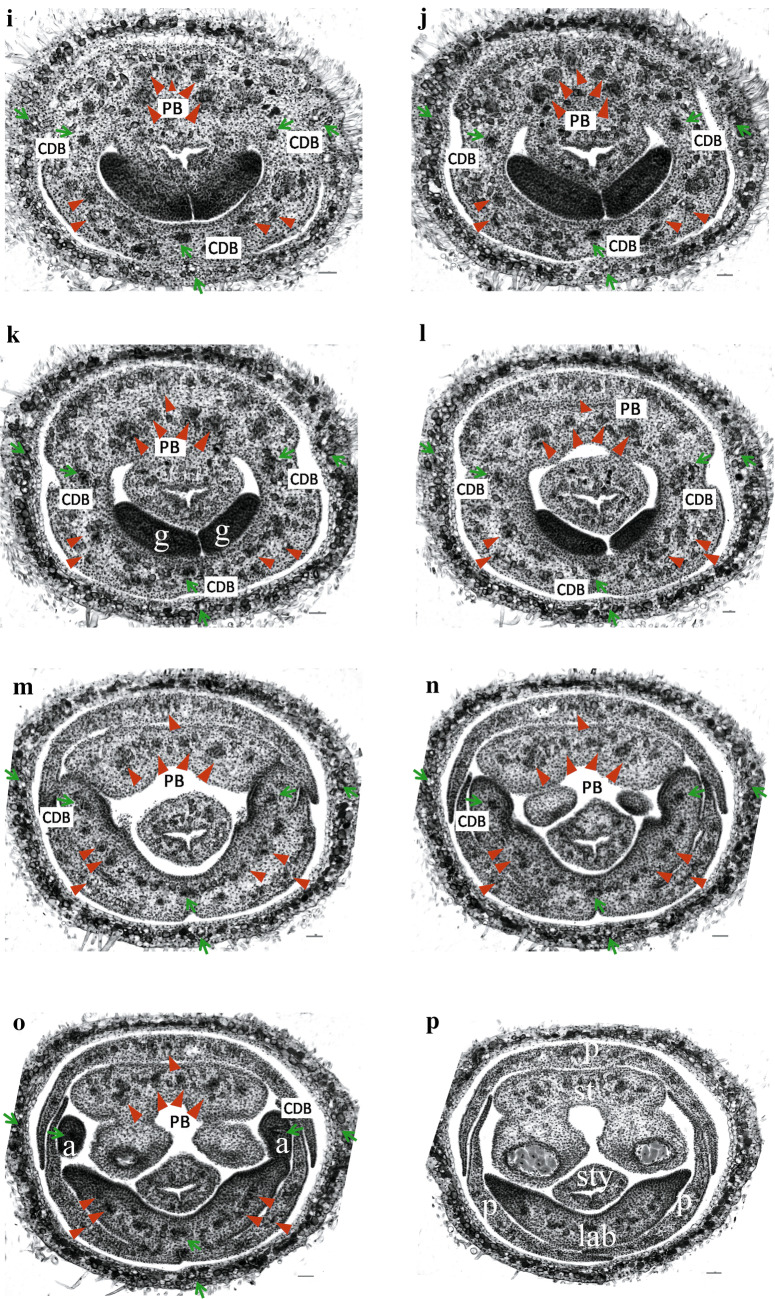
Floral vasculature in the normal flower of *A. intermeda* ‘chenshengzhen’. All figures are presented in transverse section with the adaxial side upward, related primarily to the carpellary dorsal bundles [arrow (↑), CDBs] and parietal bundles [arrowhead (▼), PBs] supply. **a** Pedicel, displaying an outer ring and an inner region of vascular bundles. **b** Sublocular region, showing the ovuliferous zone. **c** Locular region, displaying the CDBs and the PBs. **d**–**g** The upper part of the ovary locules, displaying an anastomosing vascular plexus is formed by the CDBs and PBs bearing branches each other, and merging some outer large bundles from the ovary wall. **h** Below the junction of the calyx tube, showing the calyx tube beginning to differentiate before the anastomosing vascular plexus fading out. **i**–**l** The floral tube, showing the gland emergence and petals beginning to separate. **m**–**p** A fully differentiated floral organs, showing the development of petals, labellum, fertile stamen and two appendages. Bars = 100 μm. *g* gland; *a* appendage; *s* sepal; *p* petal; *st* fertile stamen; *lab* staminodial labellum; *sty* style

**Fig. 4:**
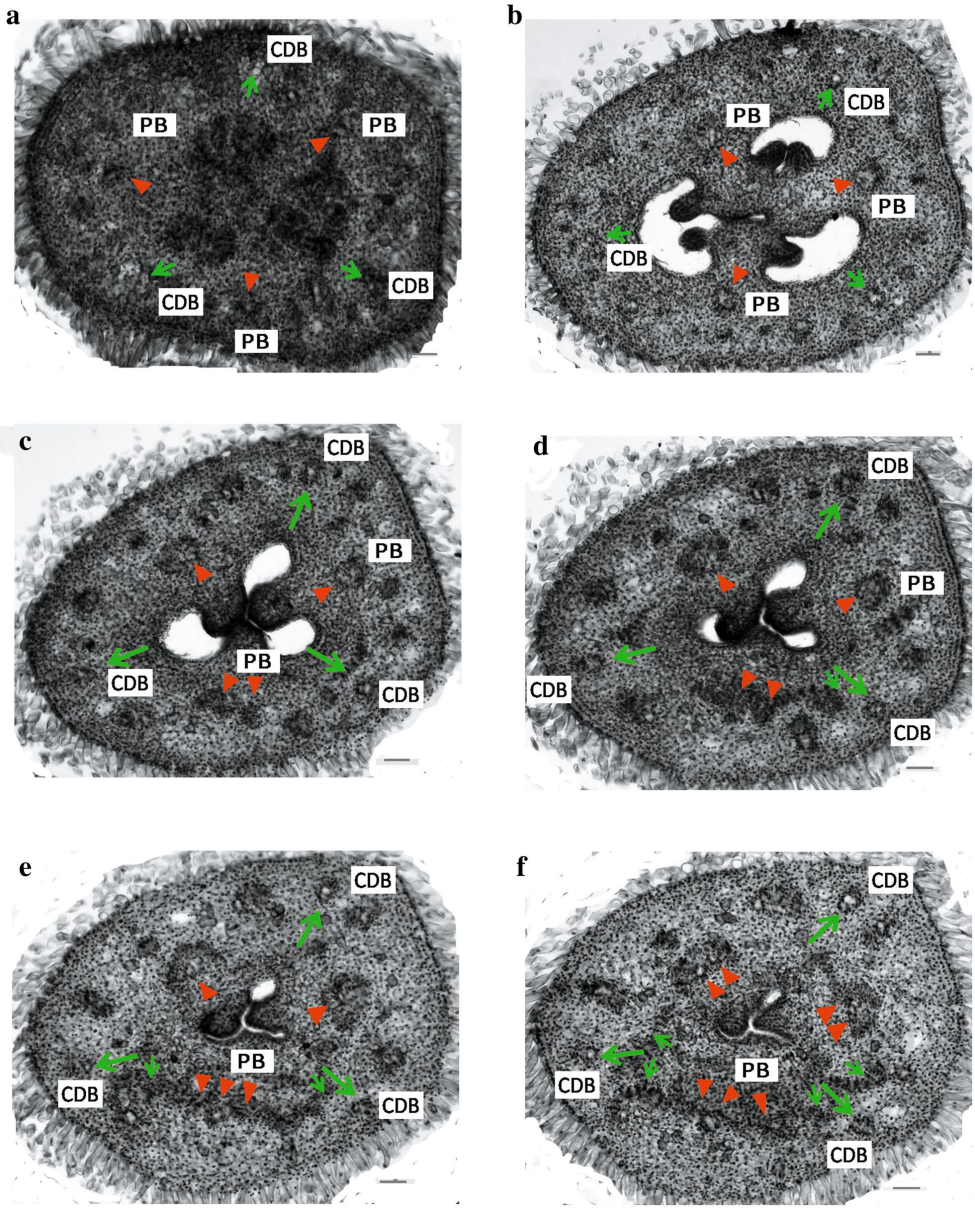

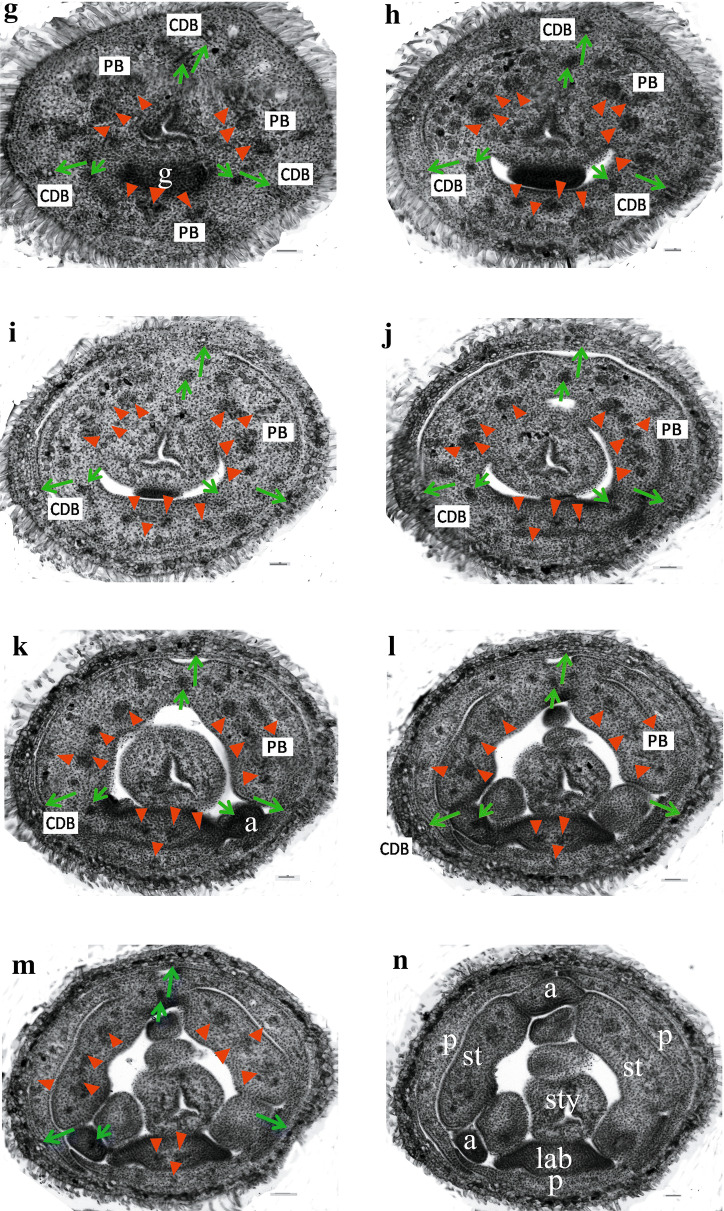
Floral vasculature in the two-staminate flower of *A. intermeda* ‘chenshengzhen’, related primarily to the carpellary dorsal bundles and parietal bundles supply. All figures are presented in transverse section with the adaxial side upward. Arrow (↑), CDB; arrowhead (▼), PB. **a** Sublocular region, showing initiating CDBs and PBs. **b** Locular region, showing invert axile placentation bearing ovules. **c**, **d** A little higher above the locular region, showing an extension of the bundles. **e**, **f** The upper part of the ovary locules, displaying the anastomosing vascular plexus being formed by the CDBs and PBs bearing branches each other, with some outer large bundles from the ovary wall also joining. , Below the junction of the calyx tube, displaying the calyx tube beginning to differentiate before **g** the anastomosing vascular plexus fading out. **h**–**j** The floral tube, showing the gland emergence and petals starting to separate. **k**–**n**, Fully differentiated floral organs, showing petals, labellum, two fertile stamens and three appendages. Bars = 100 μm. *g* gland; *a* appendage; *s* sepal; *p* petal; *st* fertile stamen; *lab* staminodial labellum; *sty* style

Serial transverse sections were taken from the normal and the two-stamen flower buds to study the vascular veins and differentiation of the parts composing the flower (Figs. 3, 4). At the floral axis beneath the ovary, a number of vascular strands distributed peripherally in two rings in the inverted trigonal pedicel. The bundles of the outer ring are arranged peripherally along the pedicel, whereas those in the inner region are irregularly arranged (Fig. 3a).

In the locular region, one is anterior, and the other two are postero–lateral among the three carpels. Hence, of forming the three septa, one septum is posterior, and the other two are antero-lateral. The locules with axile placentation appear not simultaneously but in a spiral sequence, which are in an anticlockwise way, as reported previously, e.g., Crataeva, *Alpinia clcrata*, and *Caulokaempferia coenobialis*. The three large bundles of the inner ring extend laterally and shift opposite the ovary septa as the parietal bundles (PBs) in the ovary wall. Carpellary dorsal bundles (CDBs), alternated with the 3 PBs, are oriented toward the three medians of each carpel. The remaining bundles of the inner ring anastomose into the ovary axis, forming the placental bundles (Fig. 3b). The three axile cavities fuse to form a central tri-radiate ovary fassula, which continues into the style and becomes the stylar canal. The placental bundles assemble around the ovary fassula before terminating in the base of the ovary prolongation (Fig. 3c, d).

A little higher in the locular region, the CDBs diverge outwards and divide into 2–3 branches (Fig. 3c). The inner branches of the CDBs connect with the PBs to anastomose vascular plexus in the upper part of the ovary locules, while the outer branches afterward enter the sepals and become the midribs of the sepals (Fig. 3e–g). The vascular bundles from the outer ring in the pedicel are irregularly scattered. Those large bundles join the branches of the CDBs and the PBs to produce anastomosing vascular plexus, while the small traces contribute to the sepals after the calyx tube begins to separate (Fig. 3e–g). The inner branches of the CDBs and the PBs become obscure but can be identified from their locations in the plexus.

Below the junction of the calyx tube, two antero–lateral masses of vascular bundles that are derived from the vascular plexus support the glandular emergence of the carpellary tissue (Fig. 3h). The two abaxial glands are originally attached to the inner column of the style but lie freely at a little higher within the floral tube (Fig. 3i, j). The branches of CDBs and PBs can be differentiated in their original location at the point of the plexus termination. Also, there are small traces left from the plexus, which enter the petals ultimately (Fig. 3h, k, l, m). The outer branches of the CDBs get further apart from the inner ones that would supply the two lateral appendages and the labellum (Fig. 3i, m, n).

**Fig. 5:**
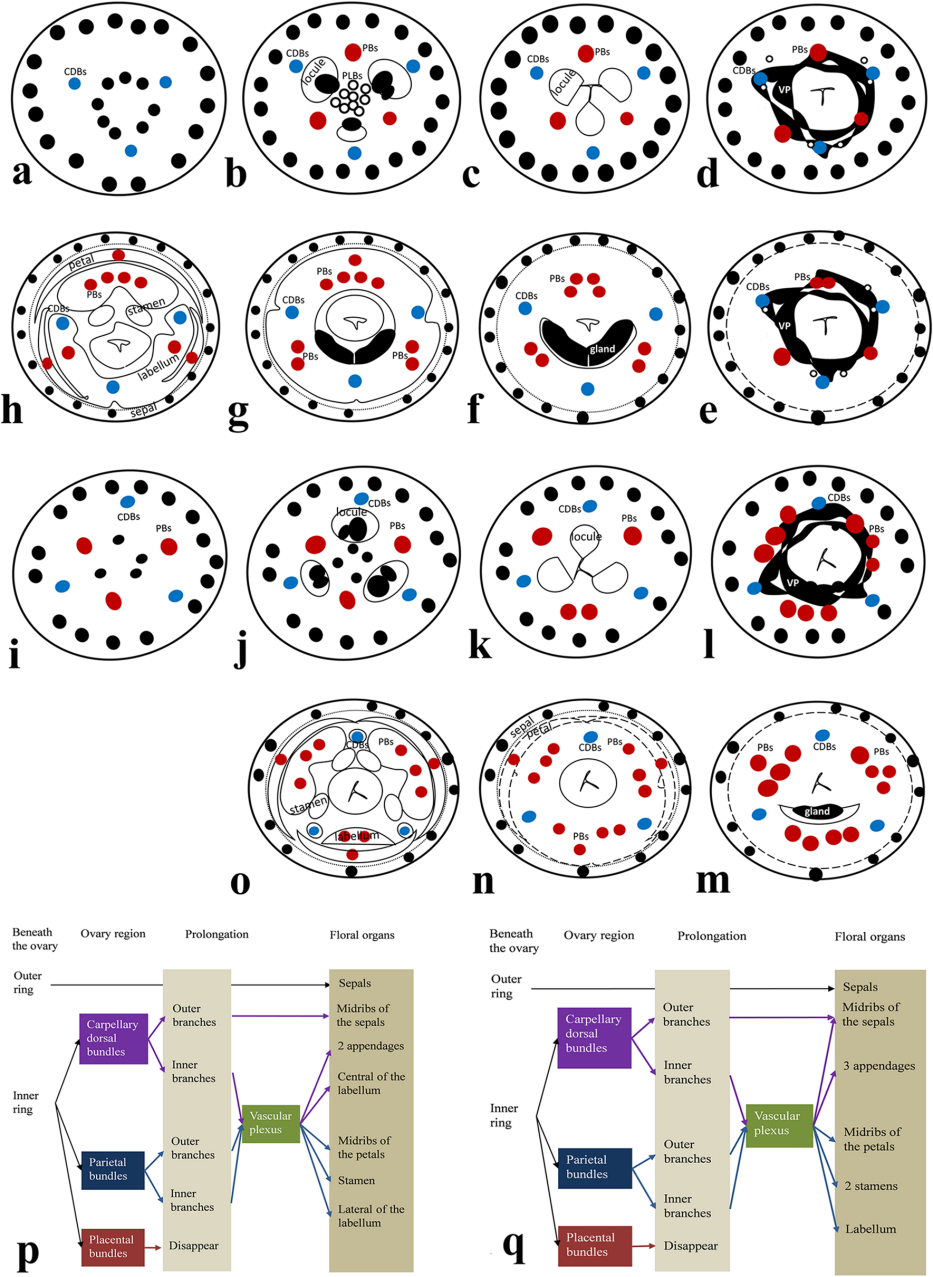
Schematic representation of the floral vasculature in *A. intermedia*. **a**–**o** Diagrams of transverse sections from the pedicel up to the style region in normal flower (**a**–**h**) and two-staminate flower (**i**–**o**). CDBs, carpel dorsal bundles, indicated in blue circles; PBs: parietal bundles, indicated in red circles; PLB: placental bundles; VP: vascular plexus; Summary of the floral vasculature in the normal flower (**p**) and the two-staminate flower (**q**). The CDBs and PBs in pedicel were distributed from the locular region where anastomose vascular plexus, to floral organs

The large adaxial (posterior) PB with two strands further divide into five strands, and two abaxial (anterolateral) PBs divide into two strands (Fig. 3i). After that, the outer strand of each PB diverges further and enters the corolla tube as the midrib of the petal when the petals are separate from the inner whorls (Fig. 3k–m). All of the four posterior parietal strands subsequently enter the fertile stamen, whereas the remaining abaxial PBs become the lateral strands of the labellum (Fig. 3m–o). The style separates from the outer column of tissues above the ovary prolongation (Fig. 3i). All the floral organs differentiate completely and can be identified (Fig. 3o). Sepals enclose all the other floral parts except the ovary, which being inferior is below it. The outmost, posterior petal embraces the other two small, antero-laterals, while the position of fertile stamen is anti-petalous. The floral vascular system in the normal flower is summarized in Fig. 5a.

At the pedicel underneath the ovary of the two-staminate flower, a number of vascular strands distributed peripherally in two rings in the inverted elliptical pedicel, the bundles of the outer ring are arranged peripherally along the pedicel, whereas those in the inner region are arranged as a triangle.

The ovary of the two-staminate flower also has three locules with axile placentation as the normal flower, but in a 180° invert way (Fig. 4a). Of the three locules, two are in the abaxial while one is in the adaxial. Three CDBs alternate with 3 PBs, respectively. Hence, the location of the CDBs and the PBs in the two-staminate flower are at the angle 180° to the normal flower. A little higher above the locular region, the abaxial PB divides horizontally to anastomose vascular plexus with inner branches of the CDBs (Fig. 4c, d). Each adaxial PB divides into two bundles which join the plexus shortly afterward (Fig. 4e, f). Some bundles of the outer ring may be connected with the plexus, but those would enter the sepals remain unaffected. The anastomosing vascular plexus is formed by the three inner branches of the CDBs and the PBs (Fig. 4f). The outer CDBs gradually go outside, enter the sepals, and become the midrib of each sepal (Fig. 4g, h). The inner dorsal bundles stay in the inner whorl until floral organs differentiate.

When the vascular plexus fades away between the top of the locular region and the bottom of the ovary prolongation, there is only a single gland at the abaxial side (Fig. 4g). The adaxial and the abaxial PBs divide into two or three and then four strands, bearing small traces (Fig. 4g–i). With the gland disappearing gradually, the outer PBs and the other daughter ones get further apart. Successively, the outer PBs go into the petals, and the other ones arrange in a line (Fig. 4j). The abaxial PBs enter the labellum, while the two adaxial ones enter the two fertile stamens (Fig. 4k, l). The inner CDBs supply to three appendages (Fig. 4g–m). The floral vascular system in the two-staminate flower is summarized in Fig. 5b.

### Floral diagrams of the normal and the two-staminate flowers

Floral diagrams showing the relative position of flower in an inflorescence, floral fusion, orientation, symmetry and structure details, have long been employed by botanists for comparative research and describing the arrangement of floral organs with great simplicity [54]. Zingiberaceae flowers frequently possess a median-adaxial (MAD) petal which is close to the floral axis and is situated away from the bract (Fig. 1). By contrast, the two-staminate flowers have a median-abaxial (MAB) petal, which is close to the bract (Fig. 1). If the two-staminate flower is considered as a 180° resupination via torsion of the ovary, the labellum, the two stamens, and the median appendage correspond to the fertile stamen, the labellum, and the absent androecial member in the normal flower, respectively (Fig. 1). The inner androecial whorl (the antepetalous whorl) consists of labellum and two stamens, and the outer androecial whorl comprised three appendages. The outer androecial bundles extend from the CDBs while the inner androecial bundles from the PBs.

### Vasculature trait based on phylogenetic analyses of Zingiberales

163 accessions, representing 45 species within the Zingiberales and 3 species within Commelinales (Additional file 1: Table S4), were included in phylogenetic analyses. We detected substitution saturation for sequences before phylogenetic reconstruction under MEGA 6 and DAMBE. For fully resolved sites, plotting rates of transitions (s) and transversions (v) against model-corrected pairwise distances revealed little saturation in MEGA and DAMBE. However, for all sites, saturation was detected by the statistical test of Xia in DAMBE (Additional file 1: Table S1, Additional file 2: Fig. S1). Phylogenetic relationships were better resolved in the combined dataset, comparing with the independent analyses of individual loci. Bayesian inference generated a topology similar to the ML tree but had higher support in some backbone branches (Additional file 2: Fig. S2). In both the ML and BI analyses, the placement of Heliconiaceae as sister to the rest of the Zingiberales consistent with the previous pattern based on ML analysis using a whole plastome data set [2]. The placement of Heliconiaceae and Musaceae is crucial in resolving relationships at the base of Zingiberales. The placement of Musaceae as sister to remaining lineages, based on maximum parsimony (MP) analysis of a combined morphological and molecular data set [30] and ML analysis of nuclear data [56], respectively, is easy to maintain, as the position requires fewer trait changes and presents a more parsimonious scenario across Zingiberales [44]. The banana clade has the same inferred ancestral character state reconstructions (ACSRs) of the floral vasculature regardless of the topology analyzed, as this clade and the relationships among its members have been consistently recovered in the three phylogenetic topologies. Thus, only the topology of the phylogenetic trees obtained from the ML analyses in this study was displayed for ancestral state reconstruction of the floral vascular system.

**Fig. 6:**
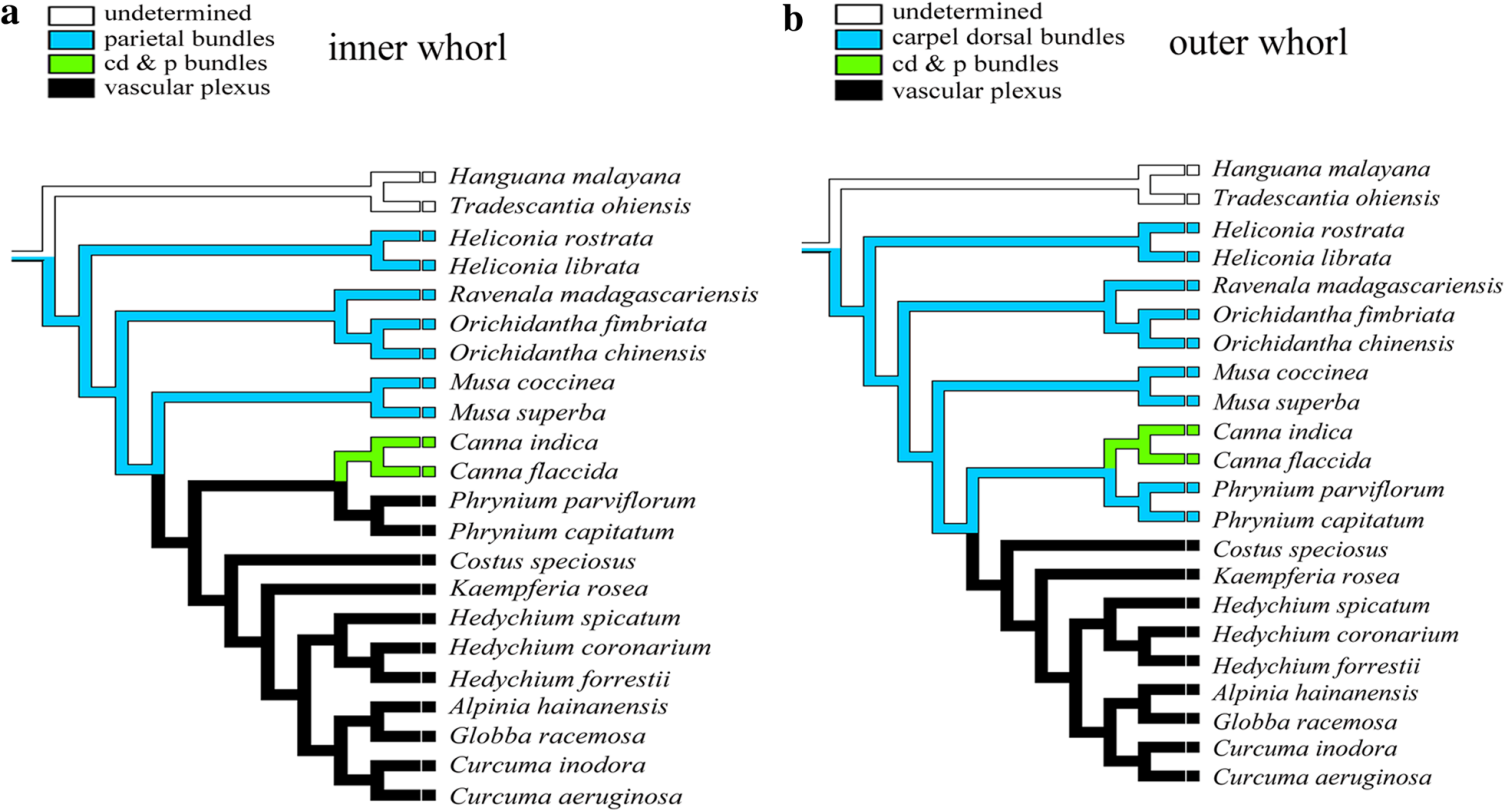
Ancestral character state reconstructions in the commelinids monocots. **a** Inner androecial whorl; **b** outer androecial member

We compared the origin of the floral vascular system in androecium to help us understand the identity of the petaloid organ in Zingiberales based on the anatomy of more than 20 species that have been studied (Additional file 1: Table S2). The outer ring of CDBs runs into the calyx as sepal midribs in many members of the order, and the androecial vasculature was associated with CDBs and PBs (Fig. 6). In the banana group, the vasculature of the inner androecial whorl has the same source, which is derived from the PBs. The outer androecial whorl is derived from the CDBs or the accompanying bundles of the dorsals (ABDs). The staminode of Heliconiaceae belongs to the outer whorl of androecium supported by the CDBs, and the staminode of the other three families is part of the inner whorl supported by the PBs. Flowers of the Musaceae family are unisexual, and the vascular system of the male flower is the same as that of the female one. The difference between them is that the locule region and the placental bundles are absent in the male flower. In the ginger group, the bundles of Zingiberaceae, Costaceae, and Marantaceae tend to anastomose vascular plexus formed by CDBs, PBs, and other bundles above the ovary. After separating into CDBs (carpellary dorsal-cure-outer staminal strands in Marantaceae) and PBs in their original location, the CDBs enter the outer androecial members while the PBs enter the inner androecial members. The androecial whorls are supplied by the CDBs and the PBs simultaneously in Cannaceae [35, 36, 64].

## Discussion

### Labellum–androecial identity

Abnormality is often observed in species of ex situ conservation and in vitro conservation, as the results of the accumulation of adaptive genetic variation and mutation caused by interspecific hybridization, tissue culture, chemical treatment, radiation exposure, parasitism and climatic change during acclimatization. On one hand, the reduction of heritable variation reduces the ability of small populations to adapt to ex situ changing conditions, and the probability of adverse factors accumulating. On the other hand, inbreeding makes some recessive mutation appear and reduce the fitness of small populations. Nevertheless, we observed two labella of *Amomum.* sp. in the wild population (unpublished data). Multiple male buds phenomenon in banana is a natural but rare occurrence, caused by the branching of inflorescence axis or the differentiation of male flowers into male buds [11]. The outer staminodes initiate and usually abort during early development in *Canna* species [39]. Many cases of homeosis are based on a phylogenetic hypothesis; environmentally induced homeosis (phenocopying) may occur through the same or similar mechanism as genetically induced homeosis [57]. The determination or circumscription of an organ is itself rather equivocal. Staminodes belonging to an androecial whorl may become secondarily petaloid in Zingiberales [5]. The labellum, a petaloid organ, blends characteristics of more petal than stamen and cannot be precisely classified as either. In comparison with other monocots, it is certain that the labellum and other petaloid appendages are homologous with stamens on the basis of their position. In the two-staminate flower, direct replacement of the fertile stamen with labellum (and vice versa) results in a 1:1 substitution. The homeotic conversion of stamen into labellum also suggests that the labellum has an androecial identity. There are about 25 to 30 flowers in one inflorescence. Apart from about 2 two-staminate flowers, we observed another two types of abnormal flowers with one fertile stamen in *A. intermedia*, 1 to 2 flowers with two labella and 1 to 2 flowers with three appendages. Song et al. [59] reported that the number of fertile stamen, labellum, appendage, and petal varied in different species of *Alpinia* genus flowers, and the relative position of fertile stamen, labellum, appendage, and petal was different from normal flowers, rotated from adaxial plane to lateral to abaxial. The continuous variation in the number of stamen may be the reappearance of the evolutionary history of the stamen in the ginger group and even in Zingiberales [59]. The number of fertile stamen from 2, 1.5, 1, 0.5 to 0, varies continuously to some extent, with intermediate phenotypes of one stamen being the most common. We speculated that the homeotic conversion occurred due to the development of common petal–stamen primordia and petal–labellum primordia were disordered during the differentiation of the floral organs. Compared with sepal and carpel, androecial members and petals show much wider variation in abnormal flower, which coincide with androecial petaloidy in Zingiberales. The bias of mutant frequency between stamen and petaloid staminodes hints that they suffered the selective pressures, which were the result of an evolutionary balance between producing pollen and attracting pollination. Several lineages of floral organ identity MADS-box genes have been isolated from Zingiberales to investigate their potential roles in the determination of androecial members [1, 3, 15, 16, 34, 58, 61, 71]. Compared with model plant systems, broad expression of B-, C-class genes, on the one hand, has expanded the regulatory potential beyond their initial framework, on the other hand, may not be specific in determining petal and androecial member identity. Previous studies imply that the *AGL6* MADS-box gene is required to specify stamen development, and low expression of *AGL6* may promote petaloidy in the androecial whorl [33, 34, 71]. Most, if not all, floral organ identity genes are dosage-dependent [6, 20, 67–69, 72], and the effects of gene dosage on a range of quantitative trait variation may have driven homeosis to occur through gene duplication and loss events. Floral organ identity genes may play central roles as transcript factors to determine the type and number of the organs formed and affect the floral architecture. In addition, the interaction between AGL6 and E-class protein may play a “bridge” or “glue” role in the multimeric complexes formation to regulate floral organ determination.

### Labellum–staminode fusion

The labellum is a major morphological structure in Lowiaceae, Zingiberaceae, Costaceae, and Cannaceae, but is not homologous among families. The labellum in Lowiaceae, which is regarded as the conspicuous petal or also receives the bundle of the adaxial, outer absent androecial member, is different from the labella in the other three families, which are only derived from staminodes [36]. In Zingiberaceae, the fate of androecial member varies, develops into fertile stamen, a highly specialized structure, or complete loss. The results of developmental work on unlobed or bilobed or trilobed labellum (Fig. 1) [7, 16, 24, 27, 58, 60, 73] generally share in ontogenic aspects and present a similar pattern: the two abaxial inner androecial members are joined through intercalary growth, fusing basally, to produce (the center of) the labellum. The two adaxial outer androecial members form two lateral staminodes, developing into small teeth at the base of the labellum, or petaloid free from the labellum, or lateral lobe fused with the central lobe of labellum to form the trilobed structure. It remains ambiguous whether the anterior, abaxial outer androecial member aborts early or never initiates since the cells in this region are indistinguishable from surrounding cells and fusion of the two segments of labellum derived from common primordia may almost be congenital. Recent transcriptome on *Zingiber zerumbet*, a trilobed labellum species, has investigated the implicated molecular mechanism that boundary genes may be involved in floral organ fusion by down-regulation, thus resulting in loss of boundary between the androecial primordia [73]. The silence of B- and C-class MADS-box genes in *Nigella damascena* not only led to homeotic conversion, but also boundary shift between floral whorls [68]. This suggests that the regulatory network of boundary genes and identity genes may contribute to the fusion between androecial members in a series of abnormal flowers.

In *Alpinia calcarata*, abnormal flowers showed two stamens, a median sterile appendage and a single gland in the postero-lateral positions [45]. The explanation was that one of the antero-lateral glands has become staminiferous, the additional fertile stamen occupies the missing gland in the abnormal flower. Therefore, the labellum which is a single organ, together with the fertile stamen, forms the outer androecial whorl. Another example of abnormal flower *A. vittata* had two lateral fertile stamens and a median sterile appendage as well as a single gland [47]. The explanation was that the two fertile stamens correspond to the two lateral staminodes, and the median sterile appendage corresponded to the posterior fertile stamen of the normal flower. Then the labellum was believed as the fusion of two antero-lateral androecial members. In this study, we observed that the two-staminate flower has two stamens, two lateral appendages, a median sterile appendage, and a single gland. The gland should not belong to the androecial whorl (see below nectary), so the labellum seems to be composed of three androecial members, as the presence of a third appendage. This result supports Gregory’s view that the labellum is a triple structure. From the above, how many androecial members composing labellum depends on largely the development of staminode. The existence of contradictory data raises the question of whether the profound integration of staminode in labellum organs could have resulted in a high degree of plasticity and dynamism. From the results of anatomy, the mid-anterior bundle in the floral tube is considered significant in the morphological nature of the labellum [41]. The mid-anterior bundle, which is derived from the vascular plexus, continues for a considerable length in the labellum, or shows an early division into two strands. Both of the above two cases are recorded in the different unlobed labellum species *Elettaria* flowers of the same plant, and the latter case also exists in some other species, such as bilobed labellum species *Kaempferia scaposa* and *Curcuma amada* [41, 51]. There is also a third case that the marginal bundles instead of mid-anterior bundle, laterally on either side of the mid-anterior line, keep upwards into the two segments of the labellum, e.g., trilobed labellum species *Zingiber macrostachyum* and bilobed labellum species *Curcuma decipiens* [51]. Pai suggested that when the labellum is emarginate, the mid-anterior bundle quickly divides into two and then run into the two components. In this study, two-staminate flowers show a median apical split in the labellum (Fig. 2c), whereas the normal ones do not, but they both possess a mid-anterior bundle. According to traditional opinion, the mid-anterior bundle may expediently be interpreted either as a composite bundle which is the fusion of the marginal bundles of the two-component members of the inner androecial whorl [41], or a vestige of the abaxial outer androecial member [17]. However, the existence of the mid-anterior bundle is independent of the labellum unlobed, bilobed or trilobed irrespective of whether the abaxial outer androecial member constitutes labellum. It seems that the development of a mid-anterior bundle and its further performance in the labellum is relevant to the degree of connation of its two components [25]. The result does not refute Payer’s view that the labellum is derived from the congenital fusion of two lateral staminodes. Recently, a study in Globba revealed that a “fifth staminode” develops as part of the labellum, supporting the view of reduction and loss of the outer abaxial staminode in Zingiberaceae [21]. Overall, the labellum comprises 2–5 androecial members, 2 or 3 androecial members in unlobed or bilobed labellum, while 4 or 5 androecial members in trilobed labellum. Anatomy of abnormal flowers may not provide enough evidence for elucidating relationships of the androecial members; it has been used to make inferences about the evolutionary homologies of different plant parts and the floral structure. However, this type of reasoning should be employed cautiously, especially the reasoning is based on the single example.

### Nectary

Nectaries are universally present in Zingiberales, except in Lowiaceae where they are aborted (only left nectary slits) and in Zingiberaceae and Costaceae where they are highly transformed [22, 26, 46]. In Musaceae family, nectaries are limited to the upper part of the ovaries above the locules in female flowers, while nectaries entirely occupied the aborted ovaries in male flowers. Carpel margins fuse incompletely to develop the septal nectarines which occur in the septa of the ovary, such as in Strelitzia flower and Heliconia flower. In contrast, the carpels fuse so completely that the structure of septal nectaries is replaced by epigynous glands in many Zingiberaceous flowers. The glands of Zingiberaceae are typically two in number and are above the septa on the antero-lateral of the flower. In some, they are basally connate on the anterior side. In rare cases, the antero-lateral glands may extend to the posterior side. The gland on the posterior side becomes reduced and absent in the evolution of the Zingiberaceous flower [47]. The two basal glands were interpreted as staminodes, epidermal appendages or stylodes. Gregory regarded the glands as epidermal appendages of the ovary, and Pai suggested that glands are more deeply associated with organs of the ovary through comparative observations [17, 41]. It is very questionable that the gland is equivalent to a staminode as it is derived from septal nectaries, which still present in other families of Zingiberales. From a positional homology point of view, stamen regressive is pervasive almost across the whole Zingiberales, in which fertile stamen evolved into staminode or totally absent. If the nectary belongs to an androecial member, then it would conflict with the establishment of monocots architecture, which is based on the trimerous plan of floral construction. Therefore, the origins of glands in Zingiberaceae could not be the androecial members. In Arabidopsis, the nectary is not regulated by ABC functional genes, but is independent of any floral organogenesis gene and is position-determined [5]. It supported the independence of nectary development from the specification of floral organ identity.

### Characteristics of vasculature in Zingiberaceae

In Zingiberales, for the common MAD petal initiation, two primary patterns of floral zygomorphy are observed, which differ mainly in the configuration of the androecium [55]. In pattern 1, the suppression of the adaxial median stamen is often associated with the presence of a relatively well-differentiated MAD petal, such as in Musaceae, Lowiaceae, and Strelitziaceae. In pattern 2, one or more abaxial stamens are reduced or modified, such as in more derived Heliconiaceae and ginger families. Interestingly, pattern 2 normally occurs in taxa that are embedded within pattern 1 clades [9, 55]. The two-staminate flower seems to accord with pattern 1 and pattern 2 on account of suppression in both the adaxial and abaxial stamen. In contrast to the normal flower, variation in the two-staminate flower occurred not only in the number and arrangement of androecium, but also in the distribution of petals and ovary. It displays putative developmental rotation in floral orientation with the median-abaxial petal by torsion of the ovary. Floral organ differentiation appears to be regulated along the adaxial–abaxial radius, with stamens developing on the adaxial side and the labellum on the abaxial side. This seems to be genetically fixed, optimizing effectiveness of the labellum as a landing platform for insect pollinators. However, whether resupination occurs needs a direct evidence, especially in developmental investigation.

Based on the previous studies, the outer androecial whorl receives its vascular supply from the CDBs and the inner androecial whorl from the PBs in both the basal banana group grade and the more derived ginger clade [35, 36, 64]. The plexus, which contributes to the petals, gland(s), and androecium, is a significant anatomical development and has been universally observed in Zingiberaceae [41]. The formation of vascular plexus is a derived character after the ginger group divergence and loss in Cannaceae. Although the stamen has degraded or lost in the process of long-term evolutionary adaptation, there is still the corresponding vascular bundle system that persists in the origin position.

Although the arrangement of the organs of the normal and abnormal flowers seems very different, the vascular bundles tend to be more consistent in supplying floral organs: (1) the bundles of the outer ring in the pedicel travel out exhibiting extensive branching while they pass upward through the flower, the sepals are supplied by these small traces derived from the outer ring and the outer branches of CDBs; (2) the large bundles from the outer ring in the pedicel, along with the branches of CDBs and the PBs, form an anastomosing vascular plexus; (3) small traces left from plexus and outer branches of PBs go into the petals; (4) the fertile stamen(s) incorporate PBs; (5) appendages are supported by inner branches of CDBs. The difference is comparing to the labellum in normal flower receives abaxial (antero-lateral) PBs and inner branches of CDBs, the smaller labellum in the two-staminate flower only receives abaxial PBs.

Transcriptome and expression data from representative species in Zingiberales have provided information for studying molecular mechanisms of floral development and diversification, but what is the specific gene and how the gene regulates coordinates the relationships of the androecial members is still unclear. Precise positional transcriptome combined with microdissection and RNA-seq techniques would facilitate the understanding of the underlying mechanism of androecial primordia development.

## Conclusion

By comparing the vasculature and anatomy of normal and abnormal flower under the microscope, the type of abnormal flower we observed was one of the quantitative variations for diverse traits. The present study suggests that anatomy of abnormal flowers may not provide enough evidence for elucidating the relationships of the androecial members, awaits further comparative ontogenesis and finer transcriptome based on microdissection work in comparison with other Zingiberales. The abnormal flowers are valuable materials for studying evolutionary relationships of not only regulatory gene functions, but also developmental structures. The vascular plexus is a significant anatomical development in Zingiberaceae.

## Supplementary information

**Additional file 1: Table S1.** Tests for significant saturation of the loci matrices for Zingiberales taxon samples. **Table S2.** Publication of the floral vascular system in Zingiberales. **Table S3.** The herbarium vouchers of the Alpinia intermedia in SCBG. **Table S4.** GeneBank accession numbers for previously sequenced species used for phylogenetic analysis in this study.

**Additional file 2: Fig. S1**. Floral diagrams to illustrate researchers’ perspective about the relationship of the epigynous glands (G), labellum (L), and lateral staminodes (LS) in two androecial whorls. It is modified from Rao (1963). a, Payer’s view. b, Brown’s view. c, Gregory’s view. d, Thompson s view**. Fig. S2.** Comparison of pairwise, uncorrected transition (s) and transversion (v) distances (y-axis) with pairwise, model-corrected distances (x-axis) for the ITS, ETS, TRN, CAM and RPLN loci. **Fig. S3**. Bayesian inference generated a topology based on ITS, ETS, TRN, CAM and RPLN combine site

## Data Availability

Not applicable.

## Abbreviations

CDBs: Carpellary dorsal bundles
PBs: Parietal bundles
ABDs: Accompanying bundles of the dorsals
MAD: Median-adaxial
MAB: Median-abaxial
ML: Maximum likelihood
BI: Bayesian inferences
ITS: Internal transcribed spacers
ETS: External transcribed spacer
CaM: Calmodulin
