# Supplementary material for: Can the anatomy of abnormal flowers elucidate relationships of the androecial members in the ginger (Zingiberaceae)?
Source: EvoDevo. 2020 Jun 9;11:12. doi: 10.1186/s13227-020-00157-8 (PMC7285767; doi:10.1186/s13227-020-00157-8)

**Fig. S1**

**
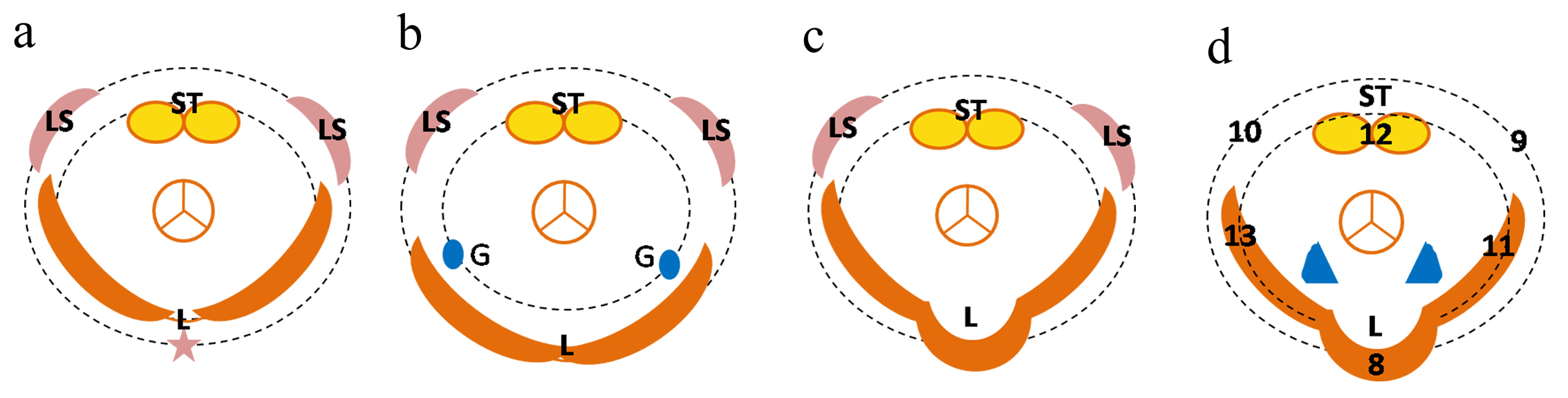
**

**Fig. S2**

**Fig.S 3**

#NEXUS

begin trees;

tree con_50_majrule = [&R] ((((((((((((Alpinia_aff[&prob=1.0,prob_stddev=0.0,prob_range={1.0,1.0},prob(percent)="100",prob+-sd="100+-0",length_mean=0.031216349,length_median=0.03110221,length_95%HPD={0.0198773,0.0414751}]:0.031102,Globba_winitii[&prob=1.0,prob_stddev=0.0,prob_range={1.0,1.0},prob(percent)="100",prob+-sd="100+-0",length_mean=0.117058543,length_median=0.1140545,length_95%HPD={0.09129876,0.149305}]:0.114055)[&prob=1.0,prob_stddev=0.0,prob_range={1.0,1.0},prob(percent)="100",prob+-sd="100+-0",length_mean=0.036956409,length_median=0.03723994,length_95%HPD={0.01862225,0.05200354}]:0.03724,((Curcuma_aeruginosa[&prob=1.0,prob_stddev=0.0,prob_range={1.0,1.0},prob(percent)="100",prob+-sd="100+-0",length_mean=0.00674015112,length_median=0.006601076,length_95%HPD={0.002786889,0.01107687}]:0.006601,Curcuma_aromatica[&prob=1.0,prob_stddev=0.0,prob_range={1.0,1.0},prob(percent)="100",prob+-sd="100+-0",length_mean=0.00457449909,length_median=0.00420956,length_95%HPD={0.001022389,0.008783392}]:0.00421)[&prob=0.998002663,prob_stddev=0.00282466091,prob_range={0.996005326,1.0},prob(percent)="100",prob+-sd="100+-0",length_mean=0.00539877541,length_median=0.005237817,length_95%HPD={5.517235E-4,0.009975951}]:0.005238,Curcuma_inodora[&prob=1.0,prob_stddev=0.0,prob_range={1.0,1.0},prob(percent)="100",prob+-sd="100+-0",length_mean=0.0400357792,length_median=0.03915956,length_95%HPD={0.02275971,0.05661336}]:0.03916,Curcuma_kwangsiensis[&prob=1.0,prob_stddev=0.0,prob_range={1.0,1.0},prob(percent)="100",prob+-sd="100+-0",length_mean=0.00293863411,length_median=0.002088173,length_95%HPD={5.623038E-6,0.008607232}]:0.002088,Curcuma_zedoaria[&prob=1.0,prob_stddev=0.0,prob_range={1.0,1.0},prob(percent)="100",prob+-sd="100+-0",length_mean=0.00506754629,length_median=0.004985213,length_95%HPD={5.439236E-5,0.009615736}]:0.004985)[&prob=1.0,prob_stddev=0.0,prob_range={1.0,1.0},prob(percent)="100",prob+-sd="100+-0",length_mean=0.0268037502,length_median=0.02673804,length_95%HPD={0.01572145,0.04005993}]:0.026738)[&prob=1.0,prob_stddev=0.0,prob_range={1.0,1.0},prob(percent)="100",prob+-sd="100+-0",length_mean=0.0516086461,length_median=0.04994075,length_95%HPD={0.02886963,0.07628806}]:0.049941,((Hedychium_coronarium[&prob=1.0,prob_stddev=0.0,prob_range={1.0,1.0},prob(percent)="100",prob+-sd="100+-0",length_mean=0.00189465453,length_median=0.001673416,length_95%HPD={4.919395E-5,0.004213581}]:0.001673,Hedychium_flavescens[&prob=1.0,prob_stddev=0.0,prob_range={1.0,1.0},prob(percent)="100",prob+-sd="100+-0",length_mean=0.00249935015,length_median=0.00216197,length_95%HPD={2.086902E-4,0.005795428}]:0.002162)[&prob=1.0,prob_stddev=0.0,prob_range={1.0,1.0},prob(percent)="100",prob+-sd="100+-0",length_mean=0.00968530311,length_median=0.009319831,length_95%HPD={0.00478712,0.01592825}]:0.00932,Hedychium_spicatum[&prob=1.0,prob_stddev=0.0,prob_range={1.0,1.0},prob(percent)="100",prob+-sd="100+-0",length_mean=0.00153511669,length_median=0.001223182,length_95%HPD={5.252929E-5,0.004195834}]:0.001223)[&prob=0.996005326,prob_stddev=0.00564932182,prob_range={0.992010652,1.0},prob(percent)="100",prob+-sd="100+-1",length_mean=0.01032402,length_median=0.01041737,length_95%HPD={0.004271248,0.01661588}]:0.010417)[&prob=1.0,prob_stddev=0.0,prob_range={1.0,1.0},prob(percent)="100",prob+-sd="100+-0",length_mean=0.0370148749,length_median=0.03767841,length_95%HPD={0.02070223,0.05415151}]:0.037678,Zingiber_spectabile[&prob=1.0,prob_stddev=0.0,prob_range={1.0,1.0},prob(percent)="100",prob+-sd="100+-0",length_mean=0.0275437276,length_median=0.02725547,length_95%HPD={0.01731478,0.03738149}]:0.027255)[&prob=0.855525965,prob_stddev=0.0555516646,prob_range={0.816245007,0.894806924},prob(percent)="86",prob+-sd="86+-6",length_mean=0.0158261107,length_median=0.01522519,length_95%HPD={0.005032469,0.02750248}]:0.015225,Zingiber_officinale[&prob=1.0,prob_stddev=0.0,prob_range={1.0,1.0},prob(percent)="100",prob+-sd="100+-0",length_mean=0.00824118053,length_median=0.007718806,length_95%HPD={5.106328E-4,0.01582866}]:0.007719)[&prob=1.0,prob_stddev=0.0,prob_range={1.0,1.0},prob(percent)="100",prob+-sd="100+-0",length_mean=0.10520736,length_median=0.1042722,length_95%HPD={0.09173316,0.1229099}]:0.104272,((Costus_pulverulentus_voucher_W[&prob=1.0,prob_stddev=0.0,prob_range={1.0,1.0},prob(percent)="100",prob+-sd="100+-0",length_mean=0.14442903,length_median=0.1440196,length_95%HPD={0.1267353,0.1606398}]:0.14402,(Dimerocostus_strobilaceus[&prob=1.0,prob_stddev=0.0,prob_range={1.0,1.0},prob(percent)="100",prob+-sd="100+-0",length_mean=0.0254603671,length_median=0.02466349,length_95%HPD={0.009071407,0.04359671}]:0.024663,Monocostus_uniflorus[&prob=1.0,prob_stddev=0.0,prob_range={1.0,1.0},prob(percent)="100",prob+-sd="100+-0",length_mean=0.00761574208,length_median=0.007483034,length_95%HPD={0.002214775,0.01164424}]:0.007483)[&prob=1.0,prob_stddev=0.0,prob_range={1.0,1.0},prob(percent)="100",prob+-sd="100+-0",length_mean=0.0254745524,length_median=0.02538642,length_95%HPD={0.01342034,0.03611721}]:0.025386)[&prob=1.0,prob_stddev=0.0,prob_range={1.0,1.0},prob(percent)="100",prob+-sd="100+-0",length_mean=0.0462556782,length_median=0.04660173,length_95%HPD={0.0308764,0.06454854}]:0.046602,Tapeinochilos_queenslandiae[&prob=1.0,prob_stddev=0.0,prob_range={1.0,1.0},prob(percent)="100",prob+-sd="100+-0",length_mean=0.073428307,length_median=0.07709136,length_95%HPD={0.01752522,0.1063341}]:0.077091)[&prob=0.906790945,prob_stddev=0.131817509,prob_range={0.813581891,1.0},prob(percent)="91",prob+-sd="91+-13",length_mean=0.0307405634,length_median=0.03044447,length_95%HPD={0.01889752,0.04713126}]:0.030444)[&prob=0.906790945,prob_stddev=0.131817509,prob_range={0.813581891,1.0},prob(percent)="91",prob+-sd="91+-13",length_mean=0.0254113964,length_median=0.02536656,length_95%HPD={0.01646566,0.03512876}]:0.025367,((Canna_indica[&prob=1.0,prob_stddev=0.0,prob_range={1.0,1.0},prob(percent)="100",prob+-sd="100+-0",length_mean=0.0710392249,length_median=0.07075295,length_95%HPD={0.05761087,0.08986213}]:0.070753,Canna_jaegeriana[&prob=1.0,prob_stddev=0.0,prob_range={1.0,1.0},prob(percent)="100",prob+-sd="100+-0",length_mean=0.0241096744,length_median=0.02364683,length_95%HPD={0.01633589,0.03347781}]:0.023647)[&prob=1.0,prob_stddev=0.0,prob_range={1.0,1.0},prob(percent)="100",prob+-sd="100+-0",length_mean=0.0716320813,length_median=0.07114631,length_95%HPD={0.05905085,0.08513862}]:0.071146,((Halopegia_azurea[&prob=1.0,prob_stddev=0.0,prob_range={1.0,1.0},prob(percent)="100",prob+-sd="100+-0",length_mean=0.0515487442,length_median=0.05127052,length_95%HPD={0.03249783,0.0647741}]:0.051271,Maranta_bicolor[&prob=1.0,prob_stddev=0.0,prob_range={1.0,1.0},prob(percent)="100",prob+-sd="100+-0",length_mean=0.0954189353,length_median=0.09544973,length_95%HPD={0.06611893,0.1340278}]:0.09545)[&prob=0.974034621,prob_stddev=0.0141233046,prob_range={0.964047936,0.984021305},prob(percent)="97",prob+-sd="97+-1",length_mean=0.0151616485,length_median=0.01447077,length_95%HPD={0.004238934,0.0298595}]:0.014471,Thaumatococcus_daniellii[&prob=1.0,prob_stddev=0.0,prob_range={1.0,1.0},prob(percent)="100",prob+-sd="100+-0",length_mean=0.327835057,length_median=0.3272432,length_95%HPD={0.2973632,0.3657521}]:0.327243)[&prob=1.0,prob_stddev=0.0,prob_range={1.0,1.0},prob(percent)="100",prob+-sd="100+-0",length_mean=0.0390922196,length_median=0.03893195,length_95%HPD={0.02472442,0.05417912}]:0.038932)[&prob=0.954061252,prob_stddev=0.0235388409,prob_range={0.937416778,0.970705726},prob(percent)="95",prob+-sd="95+-2",length_mean=0.00937520082,length_median=0.009002686,length_95%HPD={0.003323346,0.0166297}]:0.009003)[&prob=0.848868176,prob_stddev=0.208083354,prob_range={0.701731025,0.996005326},prob(percent)="85",prob+-sd="85+-21",length_mean=0.0103862042,length_median=0.01053185,length_95%HPD={0.004491123,0.0168053}]:0.010532,(((((Ensete_superbum[&prob=1.0,prob_stddev=0.0,prob_range={1.0,1.0},prob(percent)="100",prob+-sd="100+-0",length_mean=0.0208942208,length_median=0.02079945,length_95%HPD={0.01438478,0.0273738}]:0.020799,(Ensete_ventricosum[&prob=1.0,prob_stddev=0.0,prob_range={1.0,1.0},prob(percent)="100",prob+-sd="100+-0",length_mean=0.00498214049,length_median=0.004699714,length_95%HPD={0.002209138,0.008772508}]:0.0047,Musella_lasiocarpa[&prob=1.0,prob_stddev=0.0,prob_range={1.0,1.0},prob(percent)="100",prob+-sd="100+-0",length_mean=0.00450362013,length_median=0.0044117,length_95%HPD={0.001552176,0.007840648}]:0.004412)[&prob=1.0,prob_stddev=0.0,prob_range={1.0,1.0},prob(percent)="100",prob+-sd="100+-0",length_mean=0.0114549431,length_median=0.01158731,length_95%HPD={0.006514088,0.01565071}]:0.011587)[&prob=1.0,prob_stddev=0.0,prob_range={1.0,1.0},prob(percent)="100",prob+-sd="100+-0",length_mean=0.00912445695,length_median=0.009064647,length_95%HPD={0.004171645,0.01315805}]:0.009065,Musa_coccinea[&prob=1.0,prob_stddev=0.0,prob_range={1.0,1.0},prob(percent)="100",prob+-sd="100+-0",length_mean=0.0121473997,length_median=0.01191405,length_95%HPD={0.008844463,0.0161519}]:0.011914)[&prob=1.0,prob_stddev=0.0,prob_range={1.0,1.0},prob(percent)="100",prob+-sd="100+-0",length_mean=0.00923479487,length_median=0.00924764,length_95%HPD={0.005023772,0.01257574}]:0.009248,(Musa_acuminata[&prob=1.0,prob_stddev=0.0,prob_range={1.0,1.0},prob(percent)="100",prob+-sd="100+-0",length_mean=0.0128701439,length_median=0.01259491,length_95%HPD={0.009244348,0.01844921}]:0.012595,Musa_basjoo[&prob=1.0,prob_stddev=0.0,prob_range={1.0,1.0},prob(percent)="100",prob+-sd="100+-0",length_mean=0.00700295457,length_median=0.006804546,length_95%HPD={0.004529872,0.009792934}]:0.006805)[&prob=0.972703063,prob_stddev=0.01035709,prob_range={0.965379494,0.980026631},prob(percent)="97",prob+-sd="97+-1",length_mean=0.00238362361,length_median=0.002162415,length_95%HPD={1.733552E-4,0.004624367}]:0.002162)[&prob=1.0,prob_stddev=0.0,prob_range={1.0,1.0},prob(percent)="100",prob+-sd="100+-0",length_mean=0.0113099812,length_median=0.01129326,length_95%HPD={0.008133221,0.0152557}]:0.011293,Musa_ornata[&prob=1.0,prob_stddev=0.0,prob_range={1.0,1.0},prob(percent)="100",prob+-sd="100+-0",length_mean=0.0187121688,length_median=0.01848459,length_95%HPD={0.0137903,0.0240416}]:0.018485)[&prob=0.983355526,prob_stddev=0.00847398273,prob_range={0.977363515,0.989347537},prob(percent)="98",prob+-sd="98+-1",length_mean=0.00491984512,length_median=0.004767265,length_95%HPD={0.00215006,0.008087179}]:0.004767,'Musa_sp._12'[&prob=1.0,prob_stddev=0.0,prob_range={1.0,1.0},prob(percent)="100",prob+-sd="100+-0",length_mean=0.0199763892,length_median=0.01980565,length_95%HPD={0.01578271,0.02375122}]:0.019806)[&prob=1.0,prob_stddev=0.0,prob_range={1.0,1.0},prob(percent)="100",prob+-sd="100+-0",length_mean=0.0591325696,length_median=0.0590049,length_95%HPD={0.0506798,0.07033553}]:0.059005)[&prob=0.761651132,prob_stddev=0.242920838,prob_range={0.58988016,0.933422104},prob(percent)="76",prob+-sd="76+-24",length_mean=0.00385981594,length_median=0.003736586,length_95%HPD={0.001044816,0.007486242}]:0.003737,((((Orchidantha_borneensis[&prob=1.0,prob_stddev=0.0,prob_range={1.0,1.0},prob(percent)="100",prob+-sd="100+-0",length_mean=0.0140694666,length_median=0.01382194,length_95%HPD={0.005377849,0.02184086}]:0.013822,Orchidantha_chinensis[&prob=1.0,prob_stddev=0.0,prob_range={1.0,1.0},prob(percent)="100",prob+-sd="100+-0",length_mean=0.0104657015,length_median=0.01056145,length_95%HPD={0.004938494,0.01577936}]:0.010561)[&prob=0.707057257,prob_stddev=0.00941553637,prob_range={0.700399467,0.713715047},prob(percent)="71",prob+-sd="71+-1",length_mean=0.00315286694,length_median=0.00261597,length_95%HPD={3.13555E-6,0.007683638}]:0.002616,Orchidantha_maxillarioides[&prob=1.0,prob_stddev=0.0,prob_range={1.0,1.0},prob(percent)="100",prob+-sd="100+-0",length_mean=0.00528288756,length_median=0.005065311,length_95%HPD={0.001551953,0.00875342}]:0.005065)[&prob=1.0,prob_stddev=0.0,prob_range={1.0,1.0},prob(percent)="100",prob+-sd="100+-0",length_mean=0.0284940353,length_median=0.02826447,length_95%HPD={0.02082584,0.03586078}]:0.028264,Orchidantha_fimbriata[&prob=1.0,prob_stddev=0.0,prob_range={1.0,1.0},prob(percent)="100",prob+-sd="100+-0",length_mean=0.0230351266,length_median=0.02278457,length_95%HPD={0.01417188,0.03113761}]:0.022785)[&prob=1.0,prob_stddev=0.0,prob_range={1.0,1.0},prob(percent)="100",prob+-sd="100+-0",length_mean=0.0428864285,length_median=0.04302102,length_95%HPD={0.03425049,0.05186372}]:0.043021,((Ravenala_madagascariensis[&prob=1.0,prob_stddev=0.0,prob_range={1.0,1.0},prob(percent)="100",prob+-sd="100+-0",length_mean=0.00813677305,length_median=0.007914228,length_95%HPD={0.003799344,0.01234423}]:0.007914,Strelitzia_reginae[&prob=1.0,prob_stddev=0.0,prob_range={1.0,1.0},prob(percent)="100",prob+-sd="100+-0",length_mean=0.0151906734,length_median=0.01476712,length_95%HPD={0.00866804,0.02211598}]:0.014767)[&prob=0.54460719,prob_stddev=0.00564932182,prob_range={0.540612517,0.548601864},prob(percent)="54",prob+-sd="54+-1",length_mean=0.00368007002,length_median=0.003306371,length_95%HPD={7.022908E-5,0.00840163}]:0.003306,Strelitzia_caudata[&prob=1.0,prob_stddev=0.0,prob_range={1.0,1.0},prob(percent)="100",prob+-sd="100+-0",length_mean=0.0154551202,length_median=0.01498857,length_95%HPD={0.008737868,0.02304284}]:0.014989)[&prob=1.0,prob_stddev=0.0,prob_range={1.0,1.0},prob(percent)="100",prob+-sd="100+-0",length_mean=0.0150346303,length_median=0.01513822,length_95%HPD={0.008386029,0.02106143}]:0.015138)[&prob=1.0,prob_stddev=0.0,prob_range={1.0,1.0},prob(percent)="100",prob+-sd="100+-0",length_mean=0.0108325552,length_median=0.01066748,length_95%HPD={0.005749139,0.01461546}]:0.010667)[&prob=0.752330226,prob_stddev=0.241037731,prob_range={0.581890812,0.92276964},prob(percent)="75",prob+-sd="75+-24",length_mean=0.00507882124,length_median=0.004719277,length_95%HPD={6.999612E-6,0.009595093}]:0.004719,(((Heliconia_albicosta[&prob=1.0,prob_stddev=0.0,prob_range={1.0,1.0},prob(percent)="100",prob+-sd="100+-0",length_mean=0.00314719502,length_median=0.003034237,length_95%HPD={0.001206938,0.005191278}]:0.003034,Heliconia_atropurpurea[&prob=1.0,prob_stddev=0.0,prob_range={1.0,1.0},prob(percent)="100",prob+-sd="100+-0",length_mean=0.00269132476,length_median=0.002538298,length_95%HPD={9.626051E-4,0.004540369}]:0.002538)[&prob=1.0,prob_stddev=0.0,prob_range={1.0,1.0},prob(percent)="100",prob+-sd="100+-0",length_mean=0.00334800983,length_median=0.003153785,length_95%HPD={8.402365E-4,0.005994565}]:0.003154,Heliconia_wagneriana[&prob=1.0,prob_stddev=0.0,prob_range={1.0,1.0},prob(percent)="100",prob+-sd="100+-0",length_mean=0.00674041877,length_median=0.006739078,length_95%HPD={0.003592117,0.01057127}]:0.006739)[&prob=0.703062583,prob_stddev=0.0301297164,prob_range={0.681757656,0.72436751},prob(percent)="70",prob+-sd="70+-3",length_mean=0.00138228039,length_median=0.001227826,length_95%HPD={1.052786E-5,0.003303066}]:0.001228,(Heliconia_rostrata[&prob=1.0,prob_stddev=0.0,prob_range={1.0,1.0},prob(percent)="100",prob+-sd="100+-0",length_mean=0.00422262148,length_median=0.003804854,length_95%HPD={4.414791E-4,0.008259177}]:0.003805,'Heliconia_sp._Kress'[&prob=1.0,prob_stddev=0.0,prob_range={1.0,1.0},prob(percent)="100",prob+-sd="100+-0",length_mean=0.0014893233,length_median=0.001319843,length_95%HPD={3.027193E-4,0.003101874}]:0.00132)[&prob=1.0,prob_stddev=0.0,prob_range={1.0,1.0},prob(percent)="100",prob+-sd="100+-0",length_mean=0.00403150691,length_median=0.003739235,length_95%HPD={8.050252E-4,0.007647534}]:0.003739)[&prob=1.0,prob_stddev=0.0,prob_range={1.0,1.0},prob(percent)="100",prob+-sd="100+-0",length_mean=0.0246272747,length_median=0.02457284,length_95%HPD={0.01775575,0.03171675}]:0.024573)[&prob=0.800932091,prob_stddev=0.266459679,prob_range={0.612516644,0.989347537},prob(percent)="80",prob+-sd="80+-27",length_mean=0.0263287016,length_median=0.02503875,length_95%HPD={0.005560313,0.04588731}]:0.025039,(Hanguana_malayana[&prob=1.0,prob_stddev=0.0,prob_range={1.0,1.0},prob(percent)="100",prob+-sd="100+-0",length_mean=0.0772762329,length_median=0.05503475,length_95%HPD={0.01391672,0.1948866}]:0.055035,Tradescantia_ohiensis[&prob=1.0,prob_stddev=0.0,prob_range={1.0,1.0},prob(percent)="100",prob+-sd="100+-0",length_mean=0.269354689,length_median=0.2624518,length_95%HPD={0.1901255,0.42014}]:0.262452)[&prob=0.790945406,prob_stddev=0.28999852,prob_range={0.585885486,0.996005326},prob(percent)="79",prob+-sd="79+-29",length_mean=0.1307066,length_median=0.1324274,length_95%HPD={0.07815644,0.1842521}]:0.132427)[&prob=1.0,prob_stddev=0.0,prob_range={1.0,1.0},prob(percent)="100",prob+-sd="100+-0",length_mean=0.398207616,length_median=0.3949545,length_95%HPD={0.3502213,0.4584223}]:0.197477,Xiphidium_caeruleum[&prob=1.0,prob_stddev=0.0,prob_range={1.0,1.0},prob(percent)="100",prob+-sd="100+-0",length_mean=0.398207616,length_median=0.3949545,length_95%HPD={0.3502213,0.4584223}]:0.197477);

end;


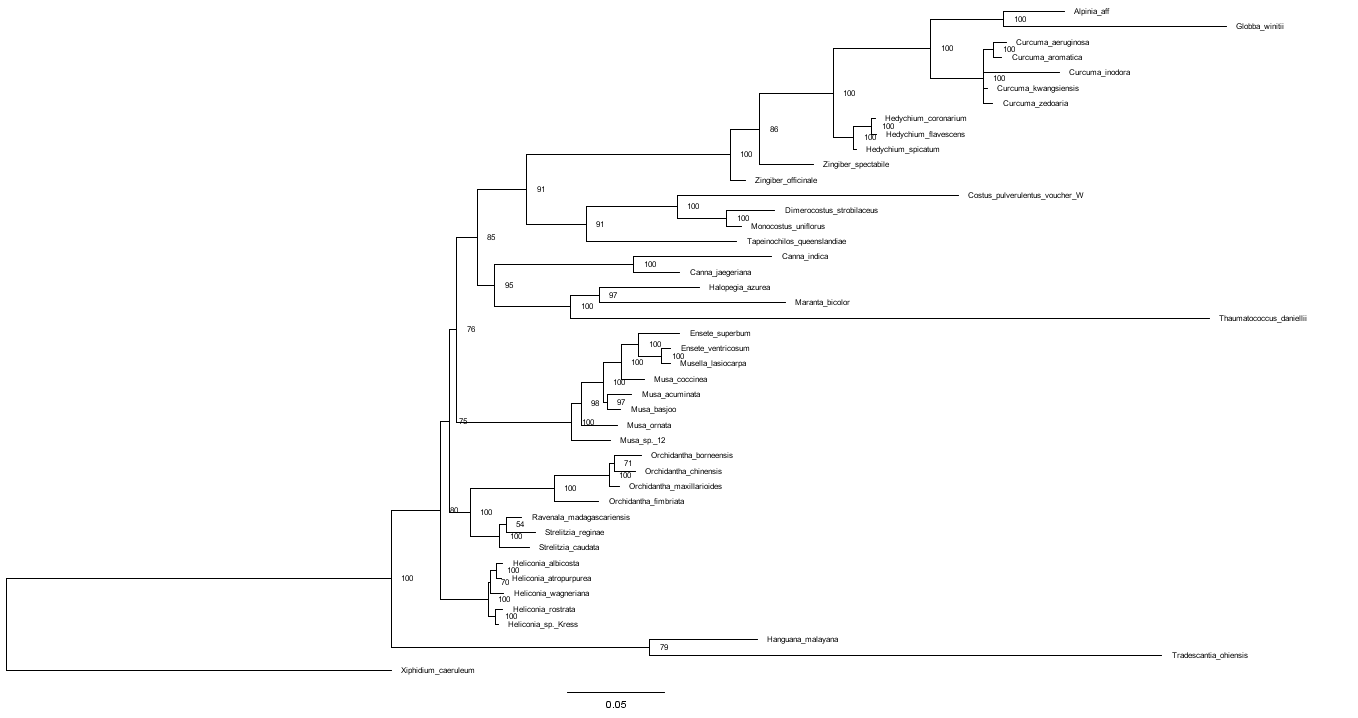

Supplement: Supplementary file 2 — Additional file 2: Fig. S1. Floral diagrams to illustrate researchers’ perspective about the relationship of the epigynous glands (G), labellum (L), and lateral staminodes (LS) in two androecial whorls. It is modified from Rao (1963). a, Payer’s view. b, Brown’s view. c, Gregory’s view. d, Thompson s view. Fig. S2. Comparison of pairwise, uncorrected transition (s) and transversion (v) distances (y-axis) with pairwise, model-corrected distances (x-axis) for the ITS, ETS, TRN, CAM and RPLN loci. Fig. S3. Bayesian inference generated a topology based on ITS, ETS, TRN, CAM and RPLN combine site [file 13227_2020_157_MOESM2_ESM.docx]
